# Supplementary material for: The Palladium(II) Complex of Aβ4−16 as Suitable Model for Structural Studies of Biorelevant Copper(II) Complexes of N-Truncated Beta-Amyloids
Source: Int J Mol Sci. 2020 Dec 2;21(23):9200. doi: 10.3390/ijms21239200 (PMC7731285; doi:10.3390/ijms21239200)
Supplement: Supplementary file 1 [file ijms-21-09200-s001.pdf]

Supporting information

Table S1. ^{1}H, and ^{13}C chemical shifts assigned for the *apo* Aβ_{4-6} peptide at 298 K on Varian Inova 500 NMR spectrometer

**Residue**	**^{1}Hα/^{13}Cα**	**^{1}Hβ/^{13}Cβ**	**Others**
Phe4	4.02 / 57.7	3.04 / 41.0	^{1}Hδ 7.16 / ^{13}Cδ 132.1<br>^{1}Hϵ 7.30 / ^{13}Cδ 131.7<br>^{1}Hζ 7.30 / ^{13}Cζ 130.3
Arg5	4.27 / 55.9	(1.70, 1.63) / 31.0	^{1}Hγ 1.46 / ^{13}Cγ 26.9<br>^{1}Hδ 3.13 / ^{13}Cδ 43.3
His6		(3.11, 3.06) / 31.0	^{1}Hδ2 7.06 / ^{13}Cδ2 120.0<br>^{1}Hϵ1 7.86 / ^{13}Cϵ1 138.5

Table S2. ^{1}H, and ^{13}C chemical shifts assigned for the Pd(Aβ_{4-6}) complex at 298 K on Varian Inova 500 NMR spectrometer

**Residue**	**^{1}Hα/^{13}Cα**	**^{1}Hβ/^{13}Cβ**	**Others**
Phe4	3.99 / 64.8	(3.15, 3.08) / 41.8	^{1}Hδ 7.35 / ^{13}Cδ 132.3<br>^{1}Hϵ 7.41 / ^{13}Cδ 131.7<br>^{1}Hζ 7.36 / ^{13}Cζ 130.1
Arg5	4.24 / 64.8	(2.00, 1.73) / 31.1	^{1}Hγ (1.60, 1.46) / ^{13}Cγ 25.0<br>^{1}Hδ 3.16 / ^{13}Cδ 44.0
His6	4.33 / 56.1	(3.23, 2.70) / 33.8	^{1}Hδ2 7.00 / ^{13}Cδ2 117.8<br>^{1}Hϵ1 7.56 / ^{13}Cϵ1 139.2

Table S3. The ^{13}C R_1 and R_2 relaxation rates extracted for the aromatic carbons in the FRH peptide acquired on natural abundance of ^{13}C isotope. The NMR experiment performed at 298 K on Varian Inova 500 NMR spectrometer

**Residue and Resonance**	R_1 **(s^{-1})**		R_2 **(s^{-1})**	
	apo	Pd(II) complex	*apo*	Pd(II) complex
Phe4 $^{13}C^{\delta}$	1.448 ± 0.006	1.495 ± 0.012	1.075 ± 0.039	1.092 ± 0.031
Phe4 $^{13}C^{\epsilon}$	1.485 ± 0.046	1.282 ± 0.024	1.126 ± 0.026	1.343 ± 0.042
His6 $^{13}C^{\delta 2}$	1.292 ± 0.030	2.635 ± 0.026	19.992 ± 3.299	4.640 ± 0.148
His6 $^{13}C^{\epsilon 1}$	1.437 ± 0.060	2.893 ± 0.162	3.041 ± 0.125	1.891 ± 0.049

Table S4. ^{1}H, ^{13}C, and ^{15}N chemical shifts assigned for apo $A\beta_{4-16}$ peptide at 298 K on Agilent DDR2 800 NMR spectrometer

**Residue**	**$^{1}H/^{15}N$** [ppm]	**$^{1}H\alpha/^{13}C\alpha$** [ppm]	**$^{1}H\beta/^{13}C\beta$** [ppm]	**Others** [ppm]
Phe4		4.17 / 57.3	(3.14, 3.08) / 40.2	$^{1}H\delta$ 7.20 / $^{13}C\delta$ 132.1<br>$^{1}H\epsilon$ 7.32 / $^{13}C\delta$ 131.7<br>$^{1}H\zeta$ 7.32 / $^{13}C\zeta$ 130.5
Arg5		4.29 / 56.0	(1.74, 1.68) / 31.2	$^{1}H\gamma$ (1.52, 1.49) / $^{13}C\gamma$ 27.0<br>$^{1}H\delta$ (3.15, 3.13) / $^{13}C\delta$ 43.3
His6		4.61 /	(3.16, 3.09) / 30.2	$^{1}H\delta2$ 7.11 / $^{13}C\delta2$ 120.1<br>$^{1}H\epsilon1$ 8.09 / $^{13}C\epsilon1$ 137.7
Asp7	8.40 / 122.0	4.64 /	2.67 / 41.3	
Ser8	8.45 / 116.5	4.39 / 59.0	(3.91, 3.87) / 63.8	
Gly9	8.55 /	(3.95, 3.87) / 45.4		
Tyr10	7.98 / 120.1	4.50 / 58.3	(3.02, 2.94) / 38.9	$^{1}H\delta$ 7.06 / $^{13}C\delta$ 133.2<br>$^{1}H\epsilon$ 6.77 / $^{13}C\epsilon$ 118.2
Glu11	8.37 / 122.6	4.20 / 56.6	(1.86, 1.92) / 30.4	$^{1}H\gamma$ (2.20, 2.15) / $^{13}C\gamma$ 36.2
Val12	8.06 / 121.2	3.93 / 62.7	1.93 / 32.6	$^{1}H\gamma1$ 0.86 / $^{13}C\gamma1$ 20.8<br>$^{1}H\gamma2$ 0.76 / $^{13}C\gamma2$ 20.8
His13	8.36 / 122.0	4.64 /	(3.11, 3.04) / 30.1	$^{1}H\delta2$ 7.08 / $^{13}C\delta2$ 119.8<br>$^{1}H\epsilon1$ 8.13 / $^{13}C\epsilon1$ 137.6
His14				
Gln15	8.50 / 122.0	4.31 / 55.9	(2.10, 1.98) / 29.5	$^{1}H\gamma$ / $^{13}C\gamma$ 33.7<br>$^{1}H\delta2$ (6.90, 7.57) / $^{15}N\delta2$ 112.6
Lys16	8.49 / 123.6	4.26 / 56.4	(1.85, 1.77) / 33.1	$^{1}H\gamma$ (1.48, 1.43) / $^{13}C\gamma$ 24.9<br>$^{1}H\delta$ 1.69 / $^{13}C\delta$ 29.1<br>$^{1}H\epsilon$ 3.00 / $^{13}C\epsilon$ 42.1

Table S5. ^{1}H, ^{13}C, and ^{15}N chemical shifts assigned for Pd(Aβ_{4-16}) complex at 298 K on Agilent DDR2 800 NMR spectrometer

**Residue**	^{1}**H**/^{15}**N** [ppm]	^{1}**H**α/^{13}**C**α [ppm]	^{1}**H**β/^{13}**C**β [ppm]	**Others** [ppm]
Phe4		4.15 / 57.3	(3.12, 3.07) / 40.3	^{1}Hδ 7.19 / ^{13}Cδ 132.1<br>^{1}Hϵ 7.31 / ^{13}Cδ 131.7<br>^{1}Hζ 7.32 / ^{13}Cζ 130.5
Arg5		4.29 / 64.9	(1.75, 1.68) / 31.2	^{1}Hγ (1.53, 1.47) / ^{13}Cγ 25.0<br>^{1}Hδ (3.15, 3.12) / ^{13}Cδ 44.0
His6		4.41 / 56.5	(3.22, 2.73) / 34.3	^{1}Hδ2 / ^{13}Cδ2<br>^{1}Hϵ1 / ^{13}Cϵ1
Asp7	8.23 / 122.3	4.43 / 52.7	(2.57, 1.84) / 40.9	
Ser8	8.13 / 115.5	4.34 / 59.1	(3.86, 3.83) / 63.7	
Gly9	8.52 / 110.6	3.88 / 45.5		
Tyr10	7.93 / 119.9	4.50 / 58.3	(3.04, 2.93) / 38.8	^{1}Hδ 7.08 / ^{13}Cδ 133.2<br>^{1}Hϵ 6.80 / ^{13}Cϵ 118.2
Glu11	8.39 / 122.6	4.20 / 56.7	(1.86, 1.93) / 30.3	^{1}Hγ (2.20, 2.16) / ^{13}Cγ 36.2
Val12	8.07 / 121.1	3.94 / 62.8	1.94 / 32.6	^{1}Hγ1 0.86 / ^{13}Cγ1 20.7<br>^{1}Hγ2 0.77 / ^{13}Cγ2 20.9
His13	8.34 / 122.0		(3.11, 3.04) / 30.1	^{1}Hδ2 7.08 / ^{13}Cδ2 119.8<br>^{1}Hϵ1 8.12 / ^{13}Cϵ1 137.6
His14			(3.14, 3.03) / 30.3	^{1}Hδ2 7.05 / ^{13}Cδ2 119.8<br>^{1}Hϵ1 8.12 / ^{13}Cϵ1 137.6
Gln15	8.49 / 122.0	4.31 / 56.0	(2.10, 1.98) / 29.4	^{1}Hγ / ^{13}Cγ 33.7<br>^{1}Hδ2 (6.90, 7.57) / ^{15}Nδ2 112.6
Lys16	8.49 / 123.6	4.26 / 56.4	(1.85, 1.77) / 33.1	^{1}Hγ (1.48, 1.43) / ^{13}Cγ 24.9<br>^{1}Hδ 1.69 / ^{13}Cδ 29.1<br>^{1}Hϵ 3.00 / ^{13}Cϵ 42.1

Table S6. The restrains for ψ and ϕ backbone and χ_1 side-chain torsion angles evaluated by TALOS-N program for apo Aβ_{4-16} peptide on base ^{1}H, ^{13}C, and ^{15}N chemical shifts.

**Residue**	ψ	ϕ	χ_1
Phe 4			
Arg 5	-70.5 ± 20.0	143.3 ± 20.5	
His 6	-85.3 ± 68.9	170.0 ± 46.8	
Asp 7	-65.3 ± 20.0	-20.7 ±64.9	-60.0 ± 30.0
Ser 8	-88.4 ± 20.0	-5.1 ± 69.7	60.0 ± 30.0
Gly 9	-87.1 ± 70.0	4.6 ± 24.1	
Tyr 10	-81.5 ± 20.6	143.9 ± 23.2	-60.0 ± 30.0
Glu 11	-65.2 ± 20.0	139.4 ± 21.2	
Val 12	-84.6 ± 42.4	135.2 ± 29.8	180.0 ± 30.0
His 13			
His 14			
Gln15			
Lys 16			

Table S7. The restrains for ψ and ϕ backbone and χ_1 side-chain torsion angles evaluated by TALOS-N program for Pd(Aβ_{4-16}) peptide on base ^{1}H, ^{13}C, and ^{15}N chemical shifts.

**Residue**	ψ	ϕ	χ_1
Phe 4			
Arg 5			
His 6			
Asp 7	-76.0 ± 20.0	137.1 ± 26.8	
Ser 8	-71.1 ± 20.0	138.8 ± 28.9	-60.0 ± 40.0
Gly 9			
Tyr 10			-60.0 ± 30.0
Glu 11	-69.2 ± 30.0	144.1 ± 45.2	-60.0 ± 40.0
Val 12	-65.8 ± 20.0	140.8 ± 20.0	180.0 ± 40.0
His 13			
His 14			
Gln15			
Lys 16			

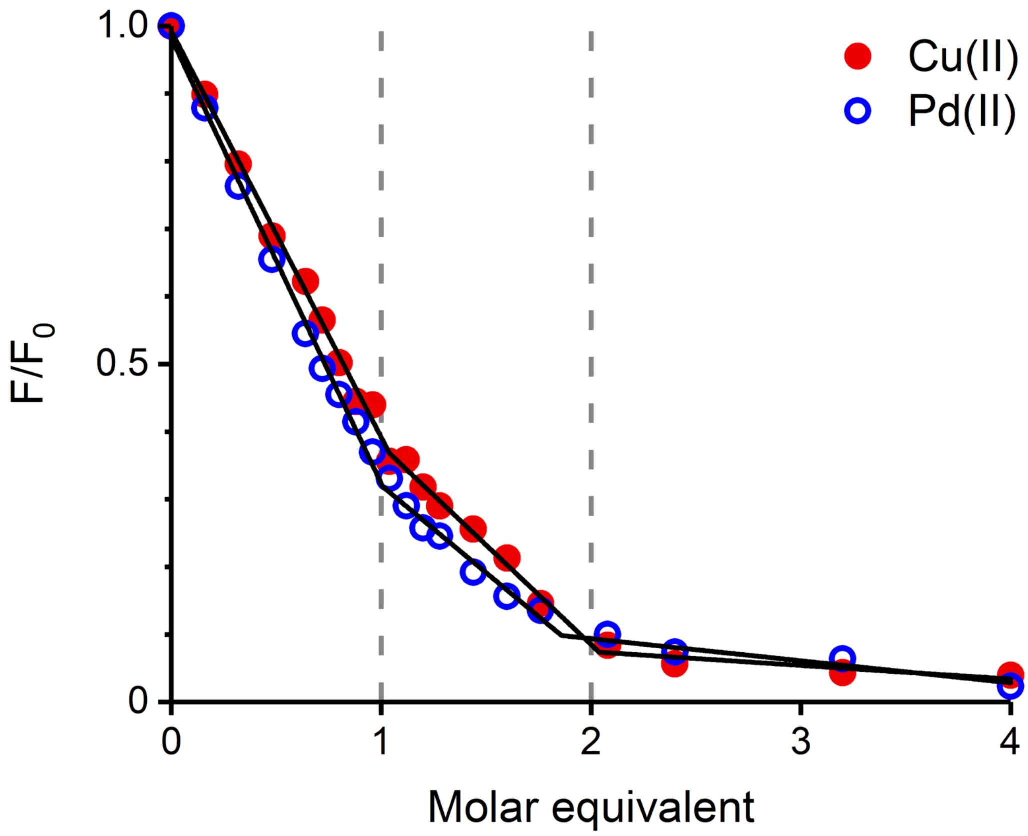

Figure S1. Aβ_{4-16} Tyr10 fluorescence (λ_{ex} = 280 nm, λ_{em} = 303 nm) quenching by Cu(II) (red dots) and Pd(II) (blue circles). Regions corresponding to the binding of the first and second metal ion equivalent are marked by dashed lines. [Aβ] = 25 μM, [HEPES] = 20 mM, pH 7.4

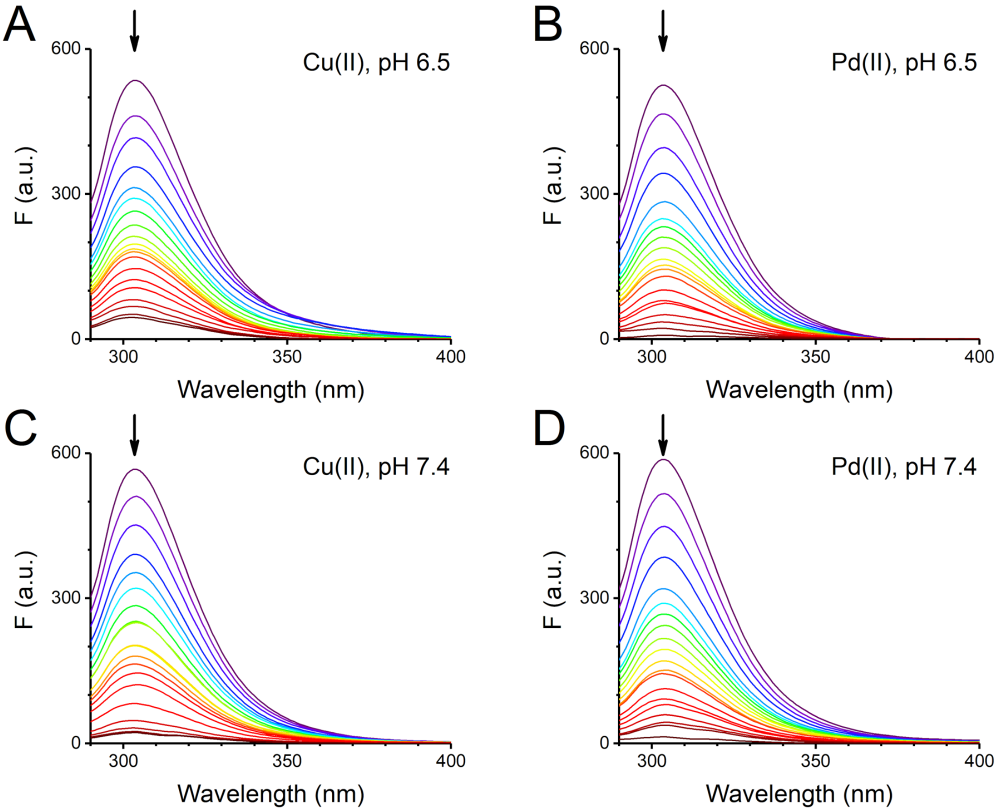

Figure S2. Aβ_{4-16} Tyr10 fluorescence (λ_{ex} = 280 nm, λ_{em} = 290-400 nm) quenching by Cu(II) (**A** and **C**) and Pd(II) (**B** and **D**). The changes were observed at pH 6.5 (20 mM MES, **A** and **B**) and 7.4 (20 mM HEPES, **C** and **D**). Shown are the spectra of the peptide with increasing concentrations of Cu(II) or Pd(II) ions. The concentration of Aβ_{4-16} was constant (25 μM), and the concentrations of metal ions were as follows: 0, 4, 8, 12, 16, 18, 20, 22, 24, 26, 28, 30, 32, 36, 40, 44, 52, 60, 80, and 100 μM.

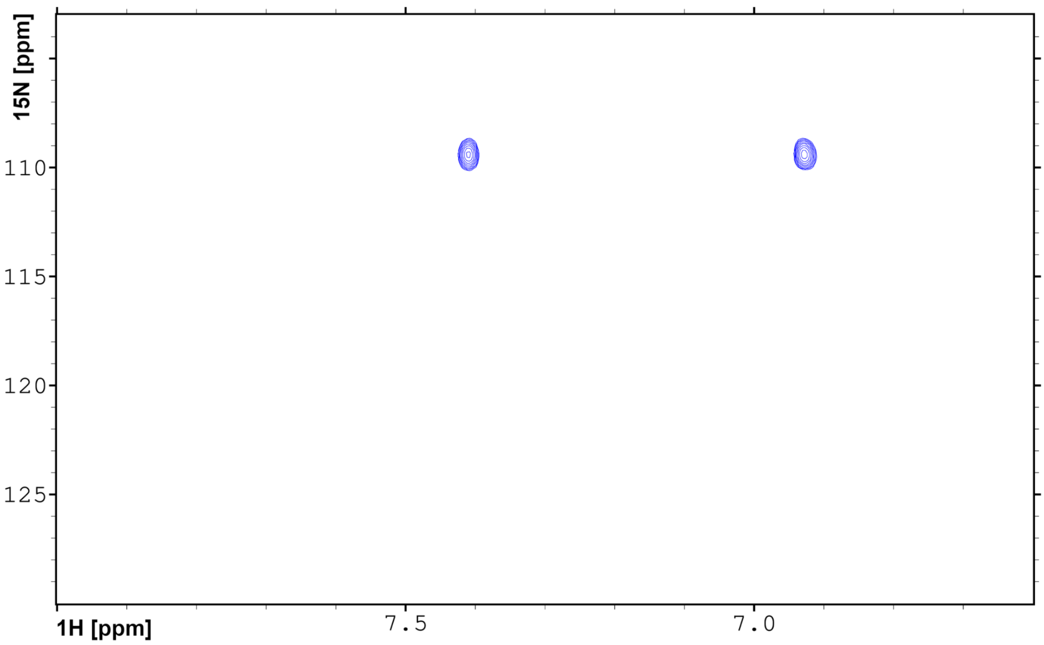

Figure S3. The ^{1}H-^{15}N HSQC spectrum acquired for Pd(Aβ_{4-6}) complex at 298 K. The experiments were performed on natural abundance of the ^{15}N isotope Varian Inova 500 NMR spectrometer.

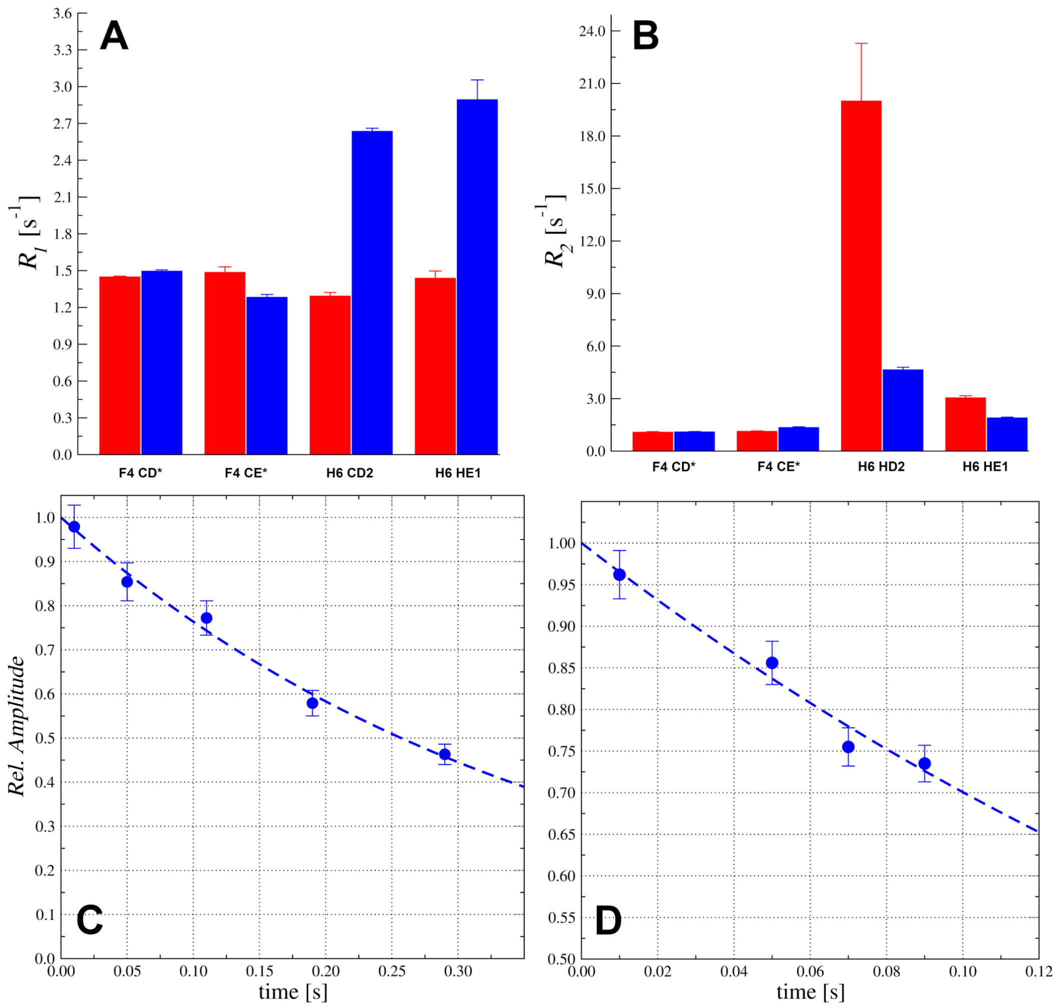

Figure S4. The values of **A)** longitudinal (R_1) and **B)** transverse (R_2) ^{13}C relaxation rates measured for aromatic carbons in Phe4 and His6 in the Aβ_{4-6} peptide for *apo* (red) and Pd(II) (blue) forms. The examples of fit relaxation data for His6 ^{13}C$^{\delta 2}$ in Aβ_{4-6} in complex with Pd(II) presented on panels **C)** and **D)** for ^{13}C R_1 and R_2 relaxation rates, respectively. The measurements were performed on the natural abundance of ^{13}C isotope utilizing on Varian Inova 500 NMR spectrometer.

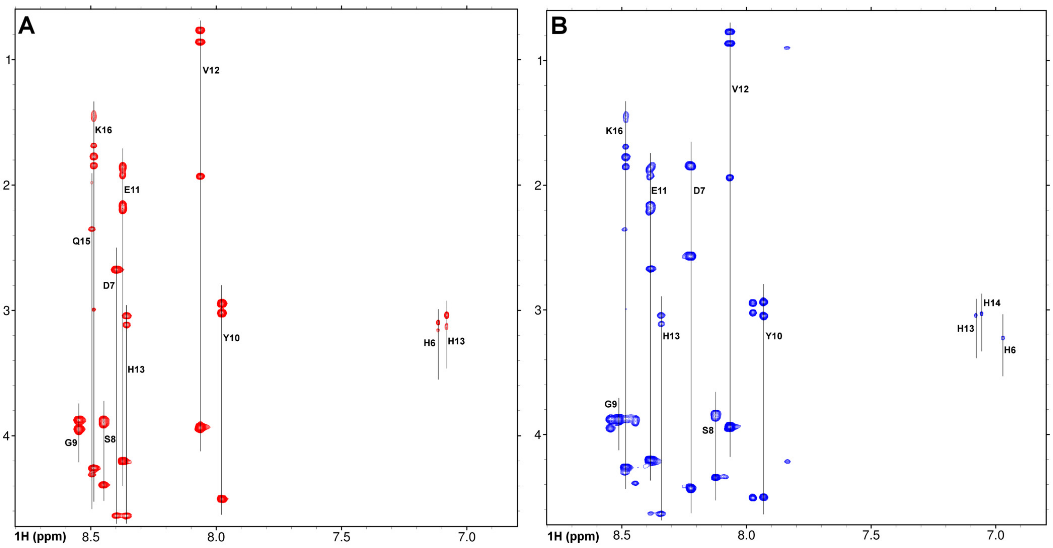

Figure S5. The amide-aliphatic part of homonuclear 2D ^{1}H-^{1}H TOCSY spectra for the Aβ_{4-16} peptide acquired with a 80 ms mixing time for *apo* **(A)** and Pd(Aβ_{4-16}) saturated **(B)** forms on an Agilent DDR2 800 NMR spectrometer at 293 K. The assignments in both forms are presented as one-letter code and sequence number. In the case of the Pd(Aβ_{4-16}) saturated form, signals (Asp7, Ser8, Gly9, Tyr10) representing the *apo* Aβ_{4-16} peptide are clearly visible. The whole assignments yielded by the analysis of NMR data are presented in Tables S4 and Table S5 for the *apo* and Pd(Aβ_{4-16}) form, respectively.

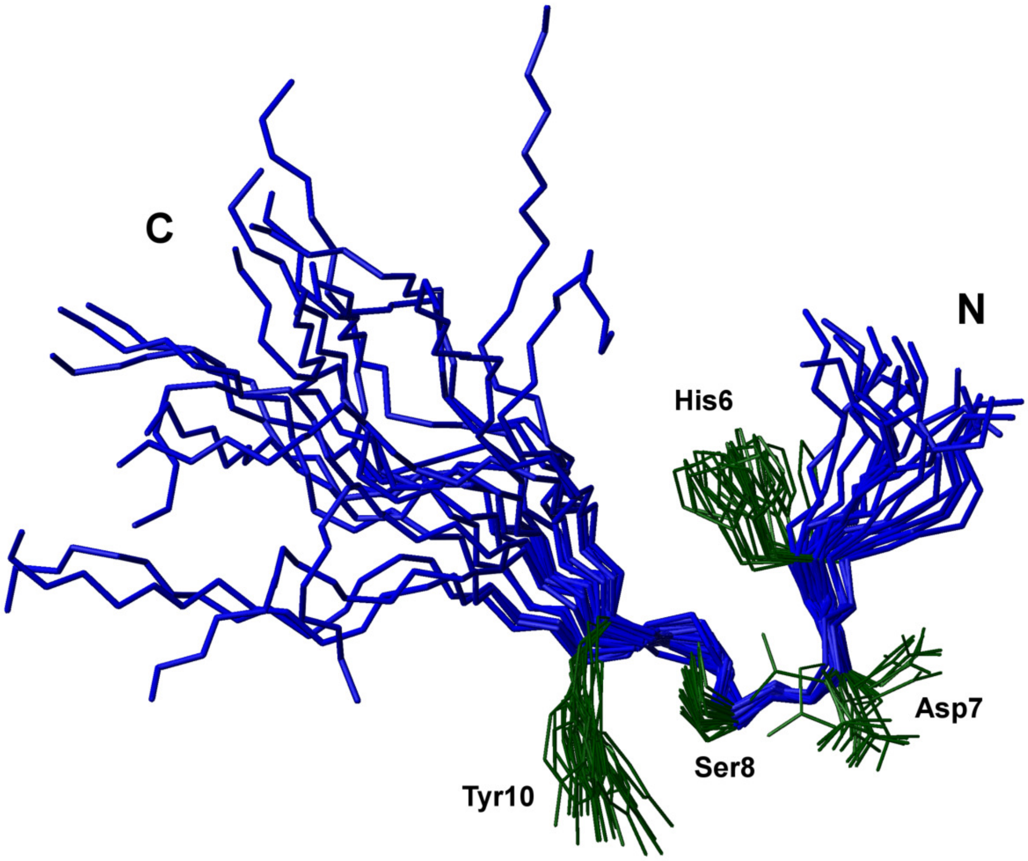

Figure S6. Ensemble of 20 low-energy structures of Aβ_{4-16} peptide in *apo* form evaluated on the base NMR data. The structures are fitted on central ^{6}HSGY^{10} motif. Orientation side-chains of the His6, Asp7, Ser8 and Tyr10 are shown in green.

© 2020 by the authors. Submitted to *Int. J. Mol. Sci.* for possible open access publication under the terms and conditions of the Creative Commons Attribution (CC BY) license (http://creativecommons.org/licenses/by/4.0/).
